# Immunoinformatics Approach to Design a Multi-Epitope Vaccine against Cutaneous Leishmaniasis

**DOI:** 10.3390/vaccines11020339

**Published:** 2023-02-02

**Authors:** Shumaila Naz, Aiman Aroosh, Ayse Caner, Esra Atalay Şahar, Seray Toz, Yusuf Ozbel, Sumra Wajid Abbasi

**Affiliations:** 1 Department of Biological Sciences, National University of Medical Sciences, Rawalpindi 46000, Pakistan; 2 Department of Parasitology, Turkey Cancer Research Center, Faculty of Medicine, Ege University, Bornova, 35100 Izmir, Turkey; 3 Department of Parasitology, Faculty of Medicine, Ege University, Bornova, 35100 Izmir, Turkey

**Keywords:** *Leishmania major*, cutaneous leishmaniasis, glycoprotein, toll-like receptor-4, molecular dynamic simulation

## Abstract

Cutaneous Leishmaniasis (CL), a neglected vector-borne disease caused by protozoan parasite Leishmania major (*L. major*), is a major public health concern, and the development of new strategies to reduce the disease incidence has become a top priority. Advances in immunoinformatics and in-silico epitope prediction could be a promising approach to designing a finest vaccine candidate. In this study, we aimed to design a peptide-based vaccine against CL using computational tools and identified ten B-cell-derived T-cell epitopes from the glycoprotein gp63 of *L. major*. All of the potential immunodominant epitopes were used to design a vaccine construct along with a linker and an adjuvant at the N-terminal for enhancing its immunogenicity. Additionally, many characteristics of the proposed vaccine were examined, and it was confirmed to be non-allergenic, non-toxic, and thermally stable. To assess the vaccine interaction with the innate immune toll-like receptor-4 (TLR-4), a 3D structure of the vaccine construct was developed. Molecular docking and molecular dynamic simulation were used to confirm the binding and to assess the stability of the vaccine-TLR4 complex and interactions, respectively. In conclusion, our multi-epitope vaccine will provide a gateway to analyze the protein function of a potential vaccine candidate against CL.

## 1. Introduction

Leishmaniasis is caused by an obligatory intracellular parasite belonging to the genus *Leishmania*, which is transmitted via the bite of infected female phlebotomine sand-flies [1]. Approximately 20 different species of the sandfly can transmit the parasite to the mammalian host, either zoonotically or anthropologically [2,3], leading to a variety of disease patterns, particularly cutaneous leishmaniasis (CL), visceral leishmaniasis (VL), and muco-cutaneous leishmaniasis (MCL) [4,5,6]. Leishmaniasis is an important global health problem [7] and the seventh most neglected tropical infection, which is prevalent in 98 countries and affects 350 million people globally [8,9,10]. 

The most commonly used drugs against CL are pentavalent antimonials, paromomycin, liposomal amphotericin B (AmBisome, AmB) and oral miltefosine [11,12,13], which have multiple adverse effects. AmB has replaced antimony as a first-line therapy for treatment, but its use is limited due to the difficulty of administration, as well as its high cost [14,15,16]. *Leishmania* (*L*.) *major*, the causative agent of zoonotic CL, expresses three main types of molecules: glycosylphosphatidylinositol, lipophosphoglycan (LPG), and glycoproteins (GP). A 63 kDa surface proteinase (GP63), a glycoprotein, was identified as the major surface antigen [17,18,19], with more than 500,000 copies expressed and distributed throughout whole promastigote cell [20]. Its role in the survival of the parasite within macrophages promotes phagocytosis and takes control over the complement activation, which increases the parasite’s resistance to complement-mediated lysis [21]. Due to its abundance and ability to develop resistance, it has been suggested that GP63 could be a candidate for the vaccine against leishmaniasis [22].

The emergence of its resistance and the increasing rate of therapeutic failures has led to the critical need for novel anti-leishmanial treatment and the development of an effective vaccine against CL [23,24]. In the last few years, immunoinformatic tools have offered epitopes predictor programs to scan the whole genomes for the immunogenic epitopes and for the selection of potential proteins for vaccine development [25,26,27]. Recently, studies of the anti-leishmanial candidates for vaccine development have advanced due to the understanding of the cell-mediated immunological mechanisms for controlling the infection [28,29]. Minimal epitopes analogous to peptides are capable of inducing the T-cell-specific responses that are essential to eradicating the intracellular parasite [30]. Based on the understating of the mechanisms of immunology, several vaccines have been designed, but none of them have been found to have any remarkable efficacy. However, the major surface glycoprotein GP63 of *L. major* considered, a ligand involved in the interaction of the parasite with the immune system, is a potential vaccine candidate that might interact directly with the macrophages [31].

A DNA vaccine containing the GP63 protein of *L. donovani* T-cell epitopes was projected to reduce the parasite load in the liver and spleen of the tested mice [32]. The GP63 protein of *L. infantum* was also reported as a potent immuno-dominant epitope that is competent enough to induce an immune response and elicit the infection against *L. infantum* [33,34,35].

The recent advances and extensive research in vaccine designing and development have provided new insights for the *Leishmania* infection [36,37]. Moreover, it helps to design new therapeutics and epitope vaccines for the molecular targets with low cost [38] and providing a useful therapeutic tool to combat the infection [39]. The present study utilized a combination of immuno-informatics strategies to develop a subunit-epitope vaccine against CL, by obtaining antigenicity, allergenicity, as well as physiochemical properties, for the vaccine protein. To check the complex stability and binding energy, molecular docking and dynamic simulations of the vaccine constructs were also carried out. The GP63 protein of *Leishmania major* was used in this study to develop a novel vaccine construct that may help in preventing CL infection in human hosts.

## 2. Materials and Methods

### 2.1. Study Design

**Figure 1:**
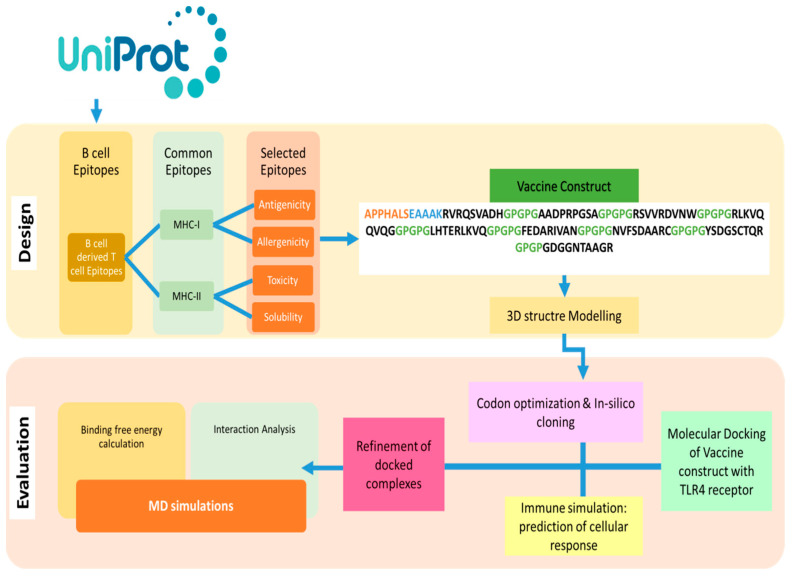
Outline of methodology followed in this study to find B and T cell epitopes and designed MEVs using immunoinformatics methods is shown in the flow chart above. Additionally, biophysical investigation was carried out using integrated docking, modeling, and binding free energies methodologies to determine the vaccine’s affinity for immunological receptors.

The design of a multi-epitope vaccine involved numerous technique steps. Figure 1 provides a summary of the general process utilized to design a multi-subunit vaccine and pipeline for the current study.

### 2.2. Sequence Retrieval and Antigenicity Prediction

The FASTA formatted full amino acid sequence of the GP63 protein (ID: P08148) from *L. major* was retrieved from Uniprot at www.uniprot.org. The antigenic nature of a protein, or its capacity to generate an immunological response within the host body, was screened using the Vaxijen 2.0 antigen prediction service (http://www.ddg-pharmfac.net/vaxijen/VaxiJen/VaxiJen.html) (accessed on 2 March 2022). This server focuses on the auto cross-covariance (ACC) transformation and alignment-independent prediction, which both retain a predictive accuracy between 70–89% [40].

### 2.3. Immunoinformatics Analysis

#### 2.3.1. B-Cell Epitope Prediction

The B-cell epitopes were predicted from the full-length protein sequences using the BCPreds method with a cutoff score of >0.8 (http://tools.iedb.org/main/bcell/) (accessed on 2 March 2022). The antigenicity of the predicted B-Cell epitopes was assessed using VaxiJen 2.0 with a threshold of 0.4 [41].

#### 2.3.2. MHC-I and MHC-II Epitopes Prediction

The CTL epitopes (9-mer) were predicted through consensus approaches, using the EDB major histocompatibility complex MHC-I binding tool (http://tools.iedb.org/mhci/) (accessed on 4 March 2022) [42]. In this investigation, the MHC allele frequency was modified using the HLA allele reference set and the suggested algorithm from IEDB 2.1 [43]. The IEDB recommended technique was utilized to predict the HTL epitopes (15-mer) using the IEDB MHC-II binding tool (https://tools.iedb.org/mhcii/) (accessed on 4 March 2022) [44].

#### 2.3.3. Epitopes Mapping

In order to determine the binding affinity potential for the dominant HLA II DRB*0101, the chosen epitopes were then employed in MHCPred 2.0. Only those with IC^50^ values of 100 nM were determined to be excellent DRB*0101 binders. After setting the cut-off to >0.6, VirulentPred and Vaxijen 2.0 was used to highlight the antigenic epitopes. Two more online servers, AllerTOP v2.0 (https://www.ddg-pharmfac.net/AllerTOP/) (accessed on 4 March 2022) [45] and ToxinPred (https://webs.iiitd.edu.in/raghava/toxinpred/protein.php) (accessed on 4 March 2022) [46], were used to check the toxicity and allergenicity, respectively, and all of the parameters were left at their default settings to ensure an 88.9% prediction accuracy. We used the established peptide affinity measurements and, as the IC^50^ values 100nM are regarded as indicating the significant affinity, we used that value to select the epitopes for further consideration [47]. Furthermore, the non-toxic epitopes were tested using the IFN-epitope server (https://webs.iiitd.edu.in/raghava/ifnepitope/index.php) (accessed on 4 March 2022).

#### 2.3.4. MEVC Designing and Post Analysis

The subunit vaccine was designed by using only the filtered epitopes, with GPGPG linkers placed at the intra-epitopic positions and APPHALS, a TLR4 peptide adjuvant, coming before it in the N-terminal and being joined by EAAK linkers [48]. The ProtParam tool of the EXPASSY server was used to examine the physiochemical characteristics of the designed MEVC and the SCRATCH protein server’s 3Dpro was used to model the three-dimensional (3D) structure of the vaccine construct from scratch [49]. Following that, loop modelling was carried out in the construct’s 3D structure using GlaxyLoop [32] from GlaxyWeb and improved using GlaxyRefine [50]. Disulphide engineering was used to improve the Design 2.0 model of the design because disulphide bonds increase the stability of the construction.

#### 2.3.5. Codon Optimization and In-Silico Cloning

Additionally, the vaccine construct’s sequence was translated in a reversible manner to optimize the codon usage for the *Escherichia coli* (*E. coli*) K12 expression system and achieve a high rate of expression. The Java Codon Adaptation Tool (JCat) (CAI) was used to calculate the expression rate of the cloned vaccine construct and was subsequently cloned using SnapGene 4.2 (https://www.snapgene.com/snapgene-viewer/) (accessed on 6 March 2022) into the *E. coli* pET28a (+) vector.

### 2.4. Molecular Docking of Vaccine with TLR4 Receptor

The minimal TLR4 was chosen as a receptor from the RCSB PDB library (PDB ID: 4G8A), and the vaccination construct was utilized as a ligand (https://www.rcsb.org/) (accessed on 6 March 2022). For molecular docking, the PatchDock server was used to evaluate the binding affinity between the designed vaccine construct and the minimized TLR4 receptor. PatchDock’s effective rigid docking technique maximizes the complementarity between geometric shapes [51]. The clustering Root Mean Square Deviation (RMSD) was left at its default value of 4.0, and the Fast Interaction Refinement in Molecular Docking (FireDock) server was used to modify the output docked solutions for the interactions [52]. The refined complex with the lowest global energy was ranked first after the refined complexes were examined.

### 2.5. Molecular Dynamics Simulation with Vaccine-TLR4 Complex

The complex (vaccine-TLR4) comprising the best vaccine construct selected in the previous phase was the only one for which a molecular dynamics simulation investigation was conducted, using the previously described methods [53]. AMBER 20 was used for the molecular dynamics simulation to evaluate and measure the protein flexibility and analysis of the intermolecular interactions was conducted using the FF14SB force filed. Additionally, several parameters, such as the RMSD (root mean square deviation), RMSF (root mean square fluctuations), salt bridges analysis, simulated trajectories, and others, were investigated to assess the complex stability.

### 2.6. Free Energy of Binding and Decomposition

Using the MMPBSA.py module of AMBER20, the free energies of the binding and per-residue free-energy decomposition were calculated [54]. The following equations were used to estimate the free binding energy of the designed complex, Gbind:

ΔG_bind, solv_ = ΔG_bind, vaccum_ + ΔG_solv, complex_ − ΔG_solv, ligand_ − ΔG_solv, complex_ (1)

ΔG_solv_ = ΔG_electrostatic(ϵ80−1)_ + ΔG_hydrophobic_ (2)

ΔG_vaccum_ = ΔE_molecular mechanics_ − T.ΔG_normal mode analysis_ (3)

The net free binding energy was decomposed into the individual residues to see which ones interacted and remained stable.

## 3. Results

### 3.1. Protein Antigenicity

The antigenic score acquired upon the running sequence of the protein through the Vaxijen 2.0 server was 0.5768, which signified that the protein is up to par immunogenicity. Hence, after checking its antigenic potential, the protein was considered to design the vaccine on the basis of its antigenic score, >0.5. The TMHMM server 2.0 transmembrane topology prediction tools predicted only one transmembrane helix for the selected protein.

### 3.2. B-Cell Epitope Prediction

**Figure 2:**
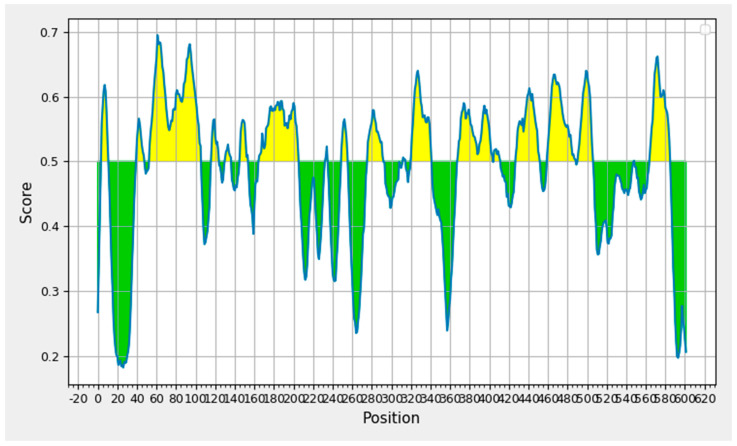
B-cell epitope prediction based on prediction results obtained through Bepipred 2.0.

The IEDB B-cell epitope prediction tool was used to predict the linear B-lymphocytes (LBL) epitopes from the chosen protein candidate GP63, and the benchmarks for the selection from the projected findings included the linear epitope, illustrated in Figure 2. Nine peptides with eight or fewer amino acid residues were disqualified from the results, yielding a total of nineteen peptide fragments. Table 1 displays the remaining nine epitope candidates. According to an antigenicity score of 1.3077, a 22-mer peptide with the sequence EVEDQGGAGSAGSHIKMRNAQD at positions 321–342 had the most antigenic potential.

### 3.3. Prediction of MHC-I and MCH-II Binding Epitopes

These B-cell peptide sequences were evaluated for the T-cell epitope prediction and the binding sites for MHC-I and MHC-II were identified. The rapid immunological response caused by the CD+ T-cells’ recognition of the MHC-I molecules on the nucleated cell surface resulted in the death of the presenting cells. On the other hand, the MHC-II molecules were found on the antigen-presenting cells (APCs) and were recognized by the CD4+ T cells. Only those epitopes that are common to both classes were taken into consideration after filtering out the MHC-III predicted epitopes based on the percentile scores and comparing them with the MHC-I allele selected epitopes. The shortened 48 common MHC-I and MHC-II epitopes were tested for antigenicity. Here, the ability of the filtered T-cell epitopes produced from the B-cells to induce and bind with the products of adaptive immunity was examined. The 29 epitopes that were produced can bind with the most common DRB*0101, with an average IC^50^ score of 34.5, a maximum of 97.5, and a minimum of 3.24. In order to remove the allergic peptides that can result in allergic reactions, the antigenic epitopes underwent allergenicity validation. Nine epitopes were non-toxic and generated IFN-gamma, while eight allergic epitopes and nineteen non-allergenic epitopes were investigated. The final set of nine epitopes obtained through various rounds of the epitope mapping phase is given in Table 2, along with the additional information that six epitopes were likely non-antigens and six demonstrated poor solubility.

### 3.4. Construction of Multi-Epitope Peptide Vaccine (MEPVC)

**Figure 3:**
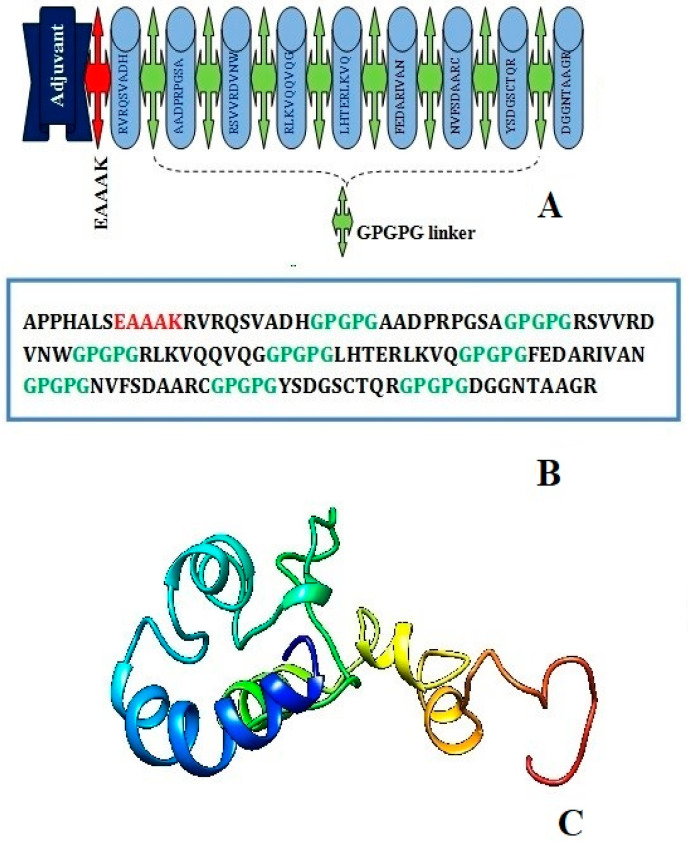
Schematic diagram of construct comprised of 119 amino acid residues; out of which first seven amino acids are TLR4 adjuvant linked with five residues of linker followed by nine immunodominant epitopes joined together by GPGPG linker (**A**) chimera sequence (**B**) and 3D structure of original predicted vaccine construct (**C**).

The EAAK linker related to the adjuvant (50S ribosomal protein L7/L12, a TLR4 agonist) at the N-terminal of the vaccine construct in order to create a stable and coherent multi-epitope peptide vaccine construct. Then, a GPGPG linker was inserted between the epitope sequences to connect the prioritized B-cell-derived T-cell epitopes. The earlier studies have emphasized that TLR4 is a member of a larger class of toll-like receptor proteins that play a critical role in initiating the cascades of immune responses against an antigen, involving both the innate immune system and the adaptive immune system, and that the EAAAK linker amplifies the bioactivity of the vaccine protein. The schematic diagram of the chimera sequence is shown in Figure 3A and the final MEV construct composed of 119 amino acids residues are represented in Figure 3B,C.

### 3.5. Antigenic and Non-Allergic Evaluation of MEPVC

The conserved predicted epitopes from the preceding steps were further analyzed for allergenicity, antigenicity, and immunogenicity properties before conceding as the potential vaccine candidates. Thus, by following the analysis, we cut off allergenic, non-antigenic and toxic epitopes and the final eight epitopes from the above list were obtained by eliminating the allergen epitope. A 9-mer epitope, DGGNTAAGR, predicted allergen was discarded from further analysis. The AllerTOP 2.0, AlgPred, and AllergenFP 1.0 servers investigated the allergenicity of the multi-epitope vaccine that was ultimately developed. According to the results of AllerTOP 2.0, the designed build does not cause inflammatory reactions. According to ANTIGENpro’s and VaxiJen’s estimates of the probability of vaccination antigenicity, the MEPVC can effectively elicit cellular and humoral immune responses against the pathogens (0.6685 and 0.5872, respectively).

### 3.6. Physiochemical Assessment and Protein Stability

The Expassy server’s ProtParam tools revealed several important features, as shown in Table 3. The molecular weight of the vaccine construct was calculated to be around 11.8 kDa, and the theoretical pI of the protein was expected to be 9.68. Size exclusion chromatography can be used to separate such small size proteins and the projected pI value showed that the vaccine construct was substantially acidic in nature. There are 13 positively charged amino acid residues and 9 negatively charged amino acid residues in total. In addition, a half-life of 4.4 h in the mammalian reticulocytes (in vitro), >20 h in the yeast (in vivo), and >10 h in the *Escherichia coli* (in vivo) were calculated. The predicted instability index (II) was 25.96, as a value less than 40 is considered to be a stable protein, and this classifies the vaccine construct as stable. The construct’s aliphatic index was found to be 51.68, indicating it is thermo-stable. A high aliphatic index indicates that the protein is stable across a wide temperature range. Its GRAVY value was calculated to be −0.682; the negative score indicated that it is hydrophilic and has better contact with the water molecules around it. The Protein-sol and Solpro servers predicted the solubility of the vaccine with a high degree of accuracy [55]. The Protein-sol calculated 0.714 and the Sol-pro calculated 0.903, indicating that the proposed MEV is more soluble upon its overexpression in *E. coli*. To summarize, the developed vaccine is expected to be extremely acidic, thermo-stable, and hydrophilic.

### 3.7. Prediction of Secondary and Tertiary Structure and Validation

According to the RaptorX Property, the MEPVC consists of 9% α-helix, 12% β-sheets, and 78% coils. The predictions demonstrated that 75% of the constituent amino acid residues were exposed, 14% were medium, and 10% were buried in terms of solvent accessibility. As no suitable template for homology modeling and threading methods was available, the 3D model of the MEPVC was created using an ab initio SCRATCH Protein Predictor.

**Figure 4:**
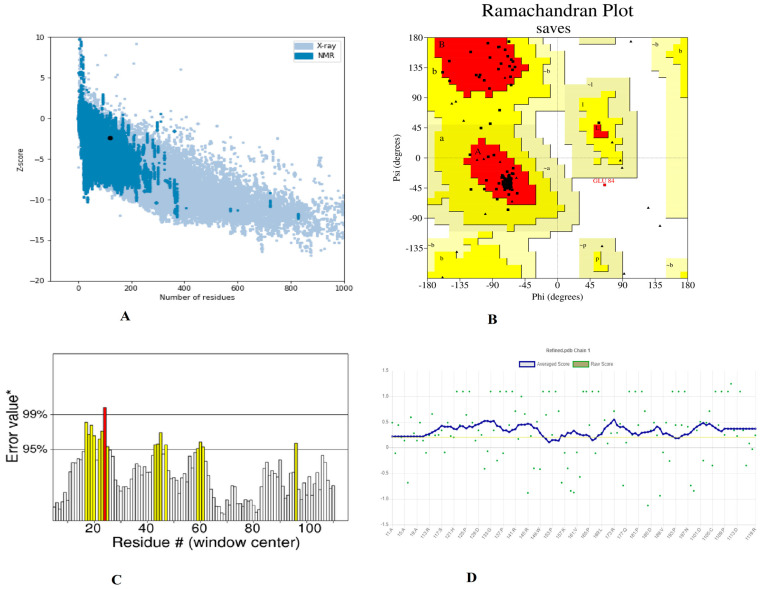
Validation of the 3D structure model of refined vaccine construct (**A**). Z-core of construct model calculated −2.37 which in range of conformation scores of native protein (**B**). Ramachandran plot validation indicates; 88%, residues are in favored, 10.7% residues in allowed and 1.3% residues in disallowed region (**C**). ERRAT factor of final construct structure was 84.90%. In ERRAT plot, gray lines are showing regions of 3D model that can be rejected at 95% confidence level and yellow lines depicting regions that can be rejected at 99% level (**D**). The 3D score of the final model was 92.44% and amino acid residues with an average 3-1D score greater than zero are regarded as reliable.

Furthermore, utilizing the GalaxyRefine server to refine a selected 3D structure of a multi-peptide vaccine, five 3D refined models were proposed. Model 5 had a higher Rama favored region (89.7) and overall acceptable GDT-HA (0.9769), RMSD (0.327), and MolProbity (2.341), as well as a lower clash score (16.5) and poor rotamers (1.2). As a result, this improved model was chosen as the best model for additional pool validation and was subjected to ProSA-web, Ramachandran Plot, and verified 3D model servers for the potential error evaluation. The refined model had a −2.37 z-score calculated through the ProSa-web, which is within a range of scores seen in native proteins of similar size (Figure 4A). According to the Ramachandran plot data, there were 66 (88%) residues in favorable, 8 (10.77%) residues in favored, 08 (10.7%) residues in allowed regions, and 1 (1.3%) residues in disallowed regions (Figure 4B). To assess the modelled structure, the ERRAT and verify 3D servers were used. The quality factor of the 3D refined model was 84.90 percent, according to the ERRAT findings (Figure 4C). The findings of the 3D score verification showed that 92.44% of the amino acid residues had a 3D-1D score >= 0.2. (Figure 4D) and in an improved 3D model, all of the residues were found to be in an acceptable side chain environment. 

### 3.8. Disulphide Engineering, Codon Optimization and In Silico Cloning Analysis

**Figure 5:**
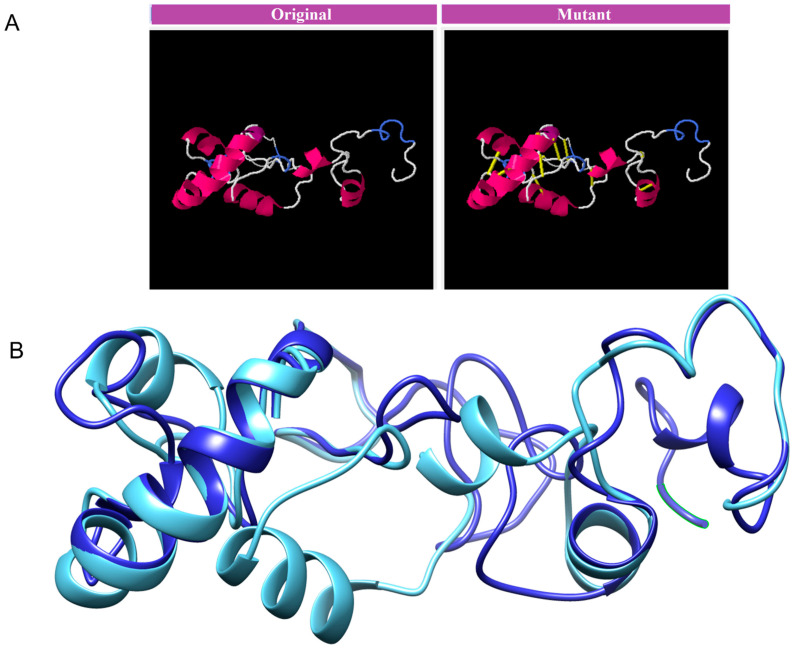
The original and mutant disulphide structures of vaccine construct (**A**). Superimposed model for the vaccine model and its mutant (**B**).

**Figure 6:**
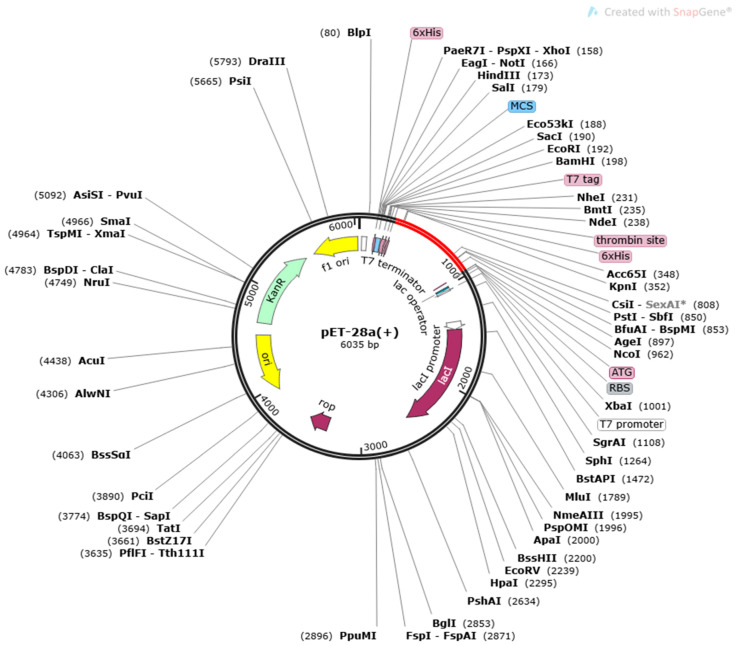
In silico cloning of a vaccine construct into the pET28a (+) vector, with the region of interest highlighted in red and surrounded by XhoI (158) and NdeI (1788), and the vector highlighted in black lines.

The MEVP was disulphide engineered to improve the molecular interactions and provide significant stability by obtaining the accurate geometric conformation. Thirteen pairs of residues were selected to be replaced with cysteine amino acids. These pairs are ALA1-GLY66 (χ^3^ angle, −65.07, energy value, 5.16 kcal/mol), PRO2-ARG45 (χ^3^ angle, +71.08, energy value, 2.6 kcal/mol), VAL14-VAL18 (χ^3^ angle, +112.54, energy value, 5.62 kcal/mol), GLY26-VAL43 (χ^3^ angle, −79.7, energy value, 2.69 kcal/mol), ALA27-GLY33 (χ^3^ angle, −107, energy value, 3.79 kcal/mol), PRO39-LYS57 (χ^3^ angle, −65.07, energy value, 5.16 kcal/mol), SER42-ARG73 (χ^3^ angle, +117.5, energy value, 4.85 kcal/mol), VAL47-GLN59 (χ^3^ angle, +98.63, energy value, 2.54 kcal/mol), PRO51-ARG55 (χ3 angle, +78.8, energy value, 2.63 kcal/mol), VAL61-GLY64 (χ^3^ angle, −114, energy value, 5.16 kcal/mol), GLY80-ASP85 (χ^3^ angle, +124.83, energy value, 4 kcal/mol), ASN91-ARG104 (χ^3^ angle, +101.81, energy value, 6.56 kcal/mol), PRO95-VAL98 (χ^3^ angle, +121.4, energy value, 2.42 kcal/mol). These residues have either a higher energy level i.e., >2 kcal/mol, or a χ^3^ angle out of range (<−79 and +71), and were selected on purpose for their stability. Disulphide bonds are a form of post-translational modification that often determines the protein structure and function. They also protect proteins against oxidants and proteolytic enzymes in extracellular environments, which can render proteins inactive. Disulfide linkages can increase the half-life of proteins and protect them from deterioration by stabilizing the proteins’ structure. Figure 5A depicts the MEPVC’s native and disulphide mutant structures. The native and mutant structures of the MEPVC were superimposed (Figure 5B), while the RMSD value for 76 pruned pairs is 0.650 Å, and across all 119 pairs, 7.128 Å. Codon optimization was then applied to the translated sequence using the JCat web server to produce a high-level protein expression in E. coli. Our optimized nucleotide sequence has a codon adaptation index (CAI) of 0.933 and a nucleotide sequence length of 64.426. These findings suggested that this optimized DNA sequence would have the highest level of expression in *E. coli* (Figure 6).

### 3.9. Docking Interaction of MEPVC and TLR4 Receptor

**Figure 7:**
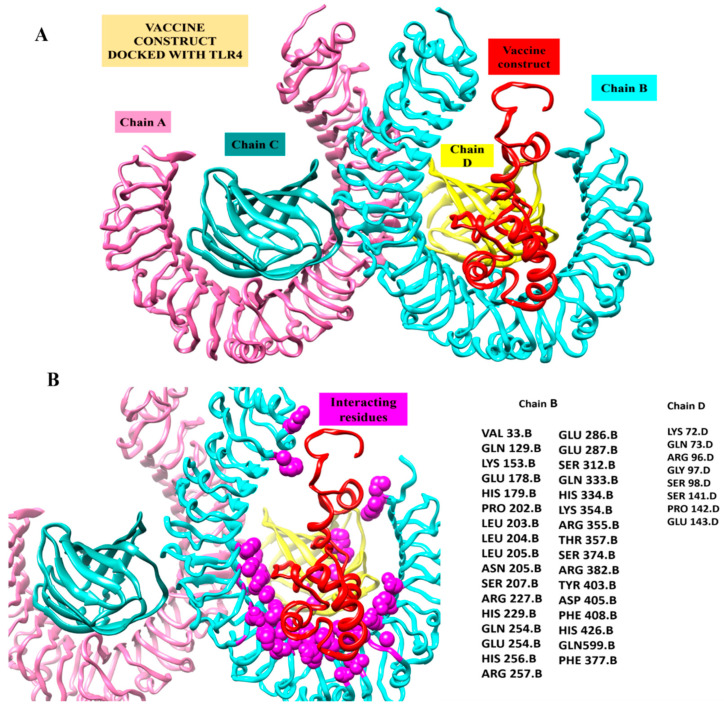
Inspection of a proposed chimeric peptide vaccine construct and the TLR4 complex using molecular docking. (**A**) Vaccine construct’s predicted docked mode in relation to TLR4. The vaccine construct is shown in red using the New-Cartoon drawing approach, while the TLR4 chains are depicted using various colored beads: Chain A (plum) Chain B (cyan) chain C (dark cyan) and Chain D (yellow). (**B**) Vaccine construct’s interactions within five Angstrom region of TLR4 Receptor. Chains B (cyan) and D (Yellow) shows interaction with Vaccine construct. The vaccine design is shown in a red cartoon, while TLR4 chain interaction residues depicted as spheres in magenta color.

In order to decipher the MEPVC’s potential for binding to induce the innate immune response, bioinformatics modeling-driven molecular docking of the proposed MEPVC to one representative innate immune response receptor (TLR4) was performed. The docking evaluation predicted the top 20 complexes, which were predominantly sorted based on the scoring function and the area size of the interacting molecules. The real rigid transformations of the complexes were then submitted to the FireDock online server for the refinement experiments. This permits a deep refinement of the predictions and makes it possible to minimize the docking procedure flexibility flaws, which lowers the possibility of false positive docking computations. With a net global energy of 8.12 kJ/mol, solution 5 was rated as having the highest level of energy. This energy is a combination of −0.57 kJ/mol hydrogen bond energy, 0.16 kJ/mol repulsive van der Waals, and −1.18 kJ/mol attractive van der Waals (vdW) (Table 4). The docked conformation of the MPEV with TLR4 and chemical interaction residues are illustrated in Figure 7. The visual assessment of the complex reveals deep MEPVC binding at the TLR4’s center, which favors weak van der Waals and rigorous hydrogen contacts with the other TLR4 residues.

### 3.10. MD Simulation Assays to Study Conformational Stability and Residual Flexibility

**Figure 8:**
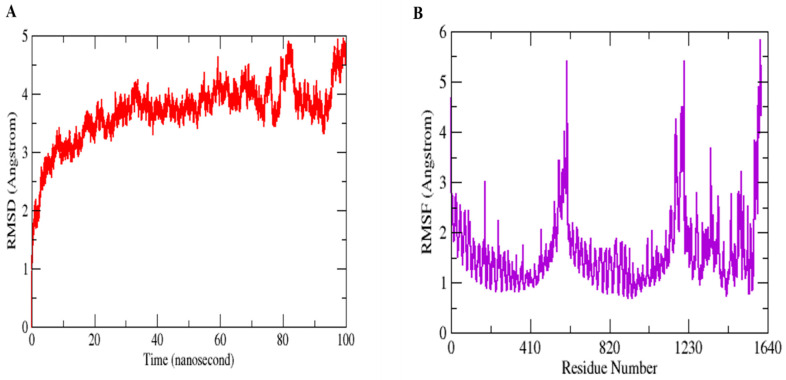
MD simulation paths are statistically analyzed. RMSD (**A**) and RMSF (**B**) are the two output values shown here.

Molecular Dynamics Simulation (MDS) is a widely used technique for examining micro-interactions between vaccine/ligand and receptor/protein complexes. To obtain a better understanding of the dynamics and stability, we ran 100 ns MD simulations of the vaccine ensemble docked-complex with TLR4 followed by RMSD and RMSF. Through a 100 ns MD simulation production run, the stability of the vaccine construct-TLR4 interaction and the complex’s dynamic behavior were clarified. Figure 8 illustrates the many statistical metrics used to assess the system stability and structural changes necessary to ensure that the vaccine construct adheres properly to the TLR4 binding site. By graphing the root mean square deviation (RMSD) over time, we were able to determine and define the complex’s conformational stability. The RMSD is the distance between the backbone carbon alpha atoms of the stacked proteins. The system exhibited a steadily growing RMSD, initially gradually rising until it reaches 40 ns, but then reached equilibrium for a short time, and the RMSD stayed uniform until 70 ns. The RMSD rose when the convergence between 80 and 100 ns was seen. However, no significant convergence indicated that the TLR4-vaccine complex is stable. Overall, the findings revealed that the complex exhibited stable behavior over the 100 ns simulation, as shown in Figure 8A. The RMSF was used to determine each complex’s residual flexibility. The residual fluctuation in the TLR4-vaccine complex was larger within residues 430–600 and 1000–1230. Increased residual fluctuation was seen in the complex. Overall, the results reveal that the docked complex has substantial behavior. Figure 8B shows the RMSFs of the complexes.

### 3.11. Determination of the Binding Free Energy of TLR4-Vaccie Ensemble Complexes

The MM-PBSA was utilized as a post-simulation processing to verify the vaccine construct’s affinity for TLR4, and the MD simulation trajectories were used to determine the molecules’ free energies in solution. As an end state free energy computation method, MM-PBSA.py was used because it is user-friendly, more accurate than docking scoring, and cheaper than free energy perturbation. The various binding free energies discovered using the GB and PB techniques are summarized in Table 5 and Table 6. The MM-PBSA analysis found that the net delta energy in GB was −272.2354 kcal/mol and in PB was −410.5471 kcal/mol. The delta energies of the complex, TLR4, and vaccine construct are −144,847.5215 kcal/mol, −109,303.9694 kcal/mol, and −35,271.3167 kcal/mol, respectively, in GB. The vaccine design contributed the most to PB (−35,207.3949 kcal/mol), followed by the complex (−144,514.7558 kcal/mol) and the TLR4 receptor (−108,896.8137 kcal/mol). In both GB and PB, the net electrostatic energy is substantially dominant and contributes favorably to the net binding energy. The system is projected to provide a net electrostatic energy contribution of −4443.1483 kal/mol to both GB and PB. In both GB and PB, the electrostatic contribution of the vaccine construct (−113,422.7858 kcal/mol) to the net PB is much greater than that of the receptor TLR4 (−81,782.776 kcal/mol) and the complex (−27,196.8614 kcal/mol). Additionally, the van der Waals energy is advantageous to the total free energy. This energy is −467.0527 kcal/mol for both GB and PB (complex = −13,331.9017 kcal/mol, TLR4 receptor = −10,091.7946 kcal/mol, vaccine construct = −2773.0544 kcal/mol). The net solvation free energy is found to be less than the total energy in both GB (4637.9657 kcal/mol) and PB (4499.654 kcal/mol), owing mostly to the polar energy (GB = 4705.7822 kcal/mol and PB = 4552.4332 kcal/mol). In comparison, non-polar salvation seems to contribute just a little amount, as GB has a −67.8165 kcal/mol and PB has a −52.7792 kcal/mol.

In order to specify the TLR4 residues that serve as a hotspot for binding or stabilizing the vaccine construct at the docked location, the net free energy of the binding in both PB and GB was further deconstructed into each TLR4 residue. For the purpose of learning more about the local interactions in a system, free energy must be decomposed. It enables the user to figure out how much each residue contributes to the net total free energy. The TLR4 and vaccine design residues in GB and PB that significantly contribute to the stability of the complex are listed in Table 7.

### 3.12. TLR4-MEPVC Stability and Salt Bridges

**Figure 9:**
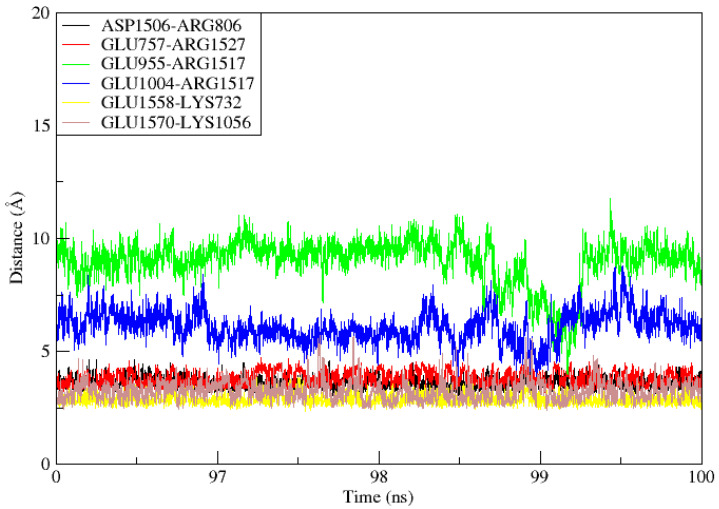
During the 100ns simulation period, salt bridges developed between TLR4 and Vaccine ensemble.

When two ionized states come into contact, salt bridges, which are non-covalent structures, arise. These interactions involve both a hydrogen bond and an electrostatic contact. In salt bridges, glutamine or aspartate serves as the acid and lysine or arginine serves as the base. The bridge is created when a proton can move from the carboxylic acid group to the amine groups of guanidine and arginine. The strongest non-covalent contacts are salt bridges, which are important for bimolecular stability. As shown in Figure 9, there were six salt bridges detected between the TLR4 (Lys732, Glu757, Arg806, Glu955, Glu1004, Lys1056) and the vaccine ensemble (Asp1506, Arg1517, Arg1527, Glu1558 and 1570 within the cut-off distance of 3.2 Å).

## 4. Discussion

The process of finding a new vaccine candidate and validating it in vitro and in vivo is costly and very time consuming [56]. However, immunoinformatics and bioinformatics technologies are now more effective than one antigen or classic deactivated pathogen vaccines and it is time saving to design multi-epitope peptide-based vaccinations [57]. Some significant studies have also demonstrated the benefits and validity of vaccines developed using these methods. These approaches are quite useful for swiftly screening antigenic vaccine compounds and many peptide-based vaccines for infectious diseases developed with immunoinformatics technologies have been experimentally confirmed and are now in use as effective vaccines [58].

In the present study, we designed a peptide-based vaccine using immunoinformatics tools against the *L. major* parasite that causes CL. Based on earlier research, we predicted several epitopes derived from the *L. major* antigenic heat shock protein, GP63. Heat shock proteins (HSPs) are intracellular proteins that are extremely conserved molecules that perform key roles in protein complex formation, protein folding, and protein translocation in parasite cells, as well as being involved in a variety of immunological processes [59]. GP63 was previously identified as a CL vaccine target in an immunoproteomics study that showed above 90% sequence similarity with various other *Leishmania* species, including *L. infantum* and *L. donovani*. On the surface of *Leishmania*, there is a zinc-dependent metalloprotease known as GP63, also known as leishmanolysin, which causes human humoral reactions [60,61]. It has been discovered that metalloproteases GP63, the main *Leishmania* surface antigen, serve a variety of vital roles in parasite’s survival. Multiple genes, whose copy counts change significantly between various species, encode GP63. One of the several vaccine possibilities being tested, primarily against CL, is Gp63 protein and numerous studies point to this surface-expressed virulence factor’s crucial function. The *L. donovani* GP63 surface protein’s possibly immunogenic T cell epitopes were designed utilizing the EpiMatrix tool kit in a study [1,12]. Additionally, in preliminary research, Gp63 antigens and a novel recombinant vaccine against *L. infantum* created using computational methods were chosen as promising immunodominant epitopes to elicit immunological responses. Hence, the current in-silico study was designed to identify the immunogenic epitopes of Gp63of *L. major* as a basis for future vaccinology studies.

In the immune system, T-cells detect peptide epitopes, which are provided by MHC molecules. These molecules are antigen-presenting cell surface proteins that are recognized by T-cell receptors (TCRs) and are divided into two classes. Almost all nucleated cells include Class I MHC molecules, which present processed proteins to CTLs via the cytosolic pathway. Class II MHC molecules, on the other hand, are present on antigen-presenting cells and represent pathogen surface proteins that are delivered to CD4+ T cells and helper T lymphocytes via the endocytic pathways [62,63]. We predicted B-cells-derived T-cells that promote the humoral and cellular immune system. Based on the structural characteristics and physiochemical properties, the immunoinformatics study revealed that our proposed multi-epitope vaccine has a large number of high affinities epitopes. Although multi-epitope vaccines offer many advantages, their most considerable disadvantage is their low immunogenicity [64]. To solve this problem, adjuvants are added to the N-terminal of the construct involved in immune system stimulation. However, several clinical patterns suggest that the T-cell responses, particularly the Th1 effector mechanisms, appear to be involved in the acquired resistance to the leishmaniasis.

Consequently, designing a successful vaccination can be possible if the suitable antigens are chosen and combined with adjuvants that stimulate a Th1 immune response [65] TLR4 is unique among the other TLRs as it engages both the MyD88/MAL and TRIF/TRAM signaling pathways and triggers TFN generation and NF-κB induction at the same time [66]. This adjuvant, on the other hand, secretes IFN- and IL2-components that fight against protozoan parasites by activating CD+ and CD8+ T-cells [67]. We also examined whether the multi-epitope peptide vaccine had a substantial affinity for the TLR4 receptor, and MD modelling confirmed the stability of the vaccine-TLR4 complex. As a result, the vaccine-TLR4 complex may trigger the TLR4-dependent signaling pathways that protect against the infection of *Leishmania*. In order to promote the bioactivity, stability and in peptide structure of the vaccine, EAAAK also linked the adjuvant TLR4 to the start point. The EAAAK linker is quite rigid, with a helical structure that is used to create and maintain space between the functional domains [68]. Linkers, which play both functional and structural roles in vaccine construction, are an important component of the multi-epitope peptide vaccine [69,70]. Additionally, GPGPG linker was used to join the assembled protein in this study. This flexible linker, which is made up of tiny non-polar amino acids similar to glycine and polar amino acids i.e., threonine and serine, was used to connect the functional domains that required inter-domain interactions. These linkers also provide flexibility and mobility to the multi-epitope vaccine construct [71].

Further, we assessed the physiochemical features, such as the toxic potential and allergic nature, of the final vaccine construct. It was found that the designed vaccine was highly antigenic, non-allergic, thermostable, and non-toxic. Thus, the secondary and the tertiary structures were investigated. The secondary structure is made up of −helix (9%), sheets (12%), and coils (78%) and the final construct’s 3D model was evaluated and confirmed to have a stable 3D structure. The interaction of the vaccination with an innate immune receptor (TLR4) was examined using this improved docking mode. TLRs are cell-surface receptors that are present on dendritic cells, macrophages, and some other immune cells and are able to recognize specific epitopic regions on parasites [72]. To combat the infection, it forms a complex and starts a downstream cascade. It has been observed that the TLR4 receptor had a considerable affinity for MEVS, and MD simulations also validated the stability of the vaccine-TLR4 complex. As a result, the vaccine-TLR4 complex may trigger the TLR4-dependent signaling pathways that protect against the infection of *Leishmania*. This in silico-developed vaccine has significant immunogenic potential and should be evaluated for an in vitro experimental study in the next phase of research. The current in silico study obtained thoroughly screened potential immunogenic epitopes for the L. major gp63 protein that could be used alone or in combination with other candidate antigens/epitopes to engineer a finely tuned, multi-epitope vaccine construct to be tested against CL in the ongoing vaccinology studies.

## 5. Conclusions

On the basis of this in silico study, future in-vitro and in-vivo studies should confirm the studied vaccine candidate’s performance in terms of the effective dose, cross-reaction, lymphocyte proliferation, cytokine production assays, as well as any potential toxicity in the relevant animal model challenges.

## Figures and Tables

**Table 1:** Selected B-cell epitopes based on linear epitope prediction method.

No.	Start	End	Peptides	Length	Antigenicity Score
**1**	40	48	HAGALQHRC	9	0.7469
**2**	54	106	QARVRQSVADHHKAPGAVSAVGLPYVTLDAAHTAAAADPRPGSARSVVRDVNW	53	0.7005
**3**	166	206	QLHTERLKVQQVQGKWKVTDMVGDICGDFKVPQAHITEGFS	41	0.5666
**4**	277	292	FEDARIVANVPNVRGK	16	0.6676
**5**	321	342	EVEDQGGAGSAGSHIKMRNAQD	22	2.1090
**6**	428	452	TRHPGLPPYWQYFTDPSLAGVSAFM	25	0.4103
**7**	460	489	PYSDGSCTQRASEAHASLLPFNVFSDAARC	30	0.8693
**8**	492	507	GAFRPKATDGIVKSYA	16	0.6250
**9**	565	585	CQGNVQAAKDGGNTAAGRRGP	21	1.4080

**Table 2:** The filtered antigenic T-cell epitopes predicted for multi subunit peptide vaccine construct.

T Cell Epitopes	Percentile Score		MHCPred Score (nM)	Allergenicity	Antigenicity	Solubility	IFN-γ	Toxicity	Virulency
	MHCI	MHCII							
RVRQSVADH	0.4	19	50.12	Non-allergen	0.6	Good soluble	+	Non-toxin	0.6586
AADPRPGSA	1.3	6.4	55.72	Non-allergen	0.8052	Good soluble	+	Non-toxin	0.6586
RSVVRDVNW	0.1	14	24.27	Non-allergen	0.9752	Good soluble	+	Non-toxin	0.6586
RLKVQQVQG	0.08	0.81	26.61	Non-allergen	0.7406	Good soluble	+	Non-toxin	0.6586
LHTERLKVQ	20	0.81	97.5	Non-allergen	0.8995	Good soluble	+	Non-toxin	0.6586
FEDARIVAN	1.3	0.73	5.93	Non-allergen	1.1664	Good soluble	+	Non-toxin	0.6586
NVFSDAARC	1.6	25	3.24	Non-allergen	1.0135	Good soluble	+	Non-toxin	0.6586
YSDGSCTQR	0.94	75	10.38	Non-allergen	0.8450	Good soluble	+	Non-toxin	0.6586
DGGNTAAGR	2.5	75.38	31.12	Non-allergen	0.9705	Good soluble	+	Non-toxin	0.6586

**Table 3:** Physiochemical properties of final vaccine construct.

Criteria	Score
**No. of amino acids**	119
**Molecular Weight**	11,825.08
**Total number of negatively charged residues**	09
**Total number of positively charged residues**	13
**Theoretical pI**	9.68
**Estimated half-life in mammalian reticulocytes in vitro**	4.4 h
**Instability Index (II)**	25.96
**Aliphatic Index**	51.68
**Grand average of hydrophaticity (GRAVY)**	−0.682
**Solubility**	0.71, 0.903

**Table 4:** Refined PatchDock complexes as an outcome of FireDock assay.

Solution Rank	Solution Number	Docking Global Energy	Attractive van der Waals Energy	Repulsive van der Waals Energy	Atomic Contact Energy	Hydrogen Bonding Energy
**1**	5	8.12	−1.18	0.16	1.74	−0.57
**2**	7	8.12	−23.52	13.15	18.17	−4.27
**3**	9	12.76	−2.38	0.64	1.29	−0.48
**4**	4	31.29	−16.52	25.75	13.70	−3.23
**5**	6	51.16	−11.81	6.06	7.43	−0.65
**6**	1	127.18	−54.05	242.23	4.95	−7.53
**7**	10	170.66	−50.85	292.63	−2.23	−5.51
**8**	2	867.90	−66.81	1157.25	12.48	−10.11
**9**	3	3487.64	−80.82	4489.93	24.81	−15.97
**10**	8	6092.43	−127.20	7856.27	16.37	−34.99

**Table 5:** Calculation of the generalized Born ESURF utilizing ‘LCPO’ surface areas. Each value is given in kcal/mol.

Generalized Born			
Complex:			
Energy Component	Average	Std. Dev.	Err. of Mean
VDWAALS	−13,331.9017	51.5473	5.1547
EEL	−113,422.7858	113.5974	11.3597
EGB	−18,564.5203	85.5735	8.5573
ESURF	471.6862	2.6545	0.2654
G gas	−126,754.6874	115.0917	11.5092
G solv	−18,092.8341	85.3047	8.5305
TOTAL	−144,847.5215	87.4204	8.742
Receptor:			
Energy Component	Average	Std. Dev.	Err. of Mean
VDWAALS	−10,091.7946	45.7379	4.5738
EEL	−81,782.776	118.6831	11.8683
EGB	−17,803.7552	89.6395	8.9639
ESURF	374.3564	2.2659	0.2266
G gas	−91,874.5705	119.4938	11.9494
G solv	−17,429.3988	88.5926	8.8593
TOTAL	−109,303.9694	82.1018	8.2102
Ligand:			
Energy Component	Average	Std. Dev.	Err. of Mean
VDWAALS	−2773.0544	20.5908	2.0591
EEL	−27,196.8614	85.3765	8.5376
EGB	−5466.5473	65.2155	6.5216
ESURF	165.1464	1.205	0.1205
G gas	−29,969.9158	83.196	8.3196
G solv	−5301.4009	65.3738	6.5374
TOTAL	−35,271.3167	42.7365	4.2737
Differences (Complex-Receptor—Ligand):			
Energy Component	Average	Std. Dev.	Err. of Mean
VDWAALS	−467.0527	10.8948	1.0895
EEL	−4443.1483	64.8796	6.488
EGB	4705.7822	56.3535	5.6354
ESURF	−67.8165	0.8586	0.0859
DELTA G gas	−4910.2011	62.5925	6.2592
DELTA G solv	4637.9657	55.8599	5.586
DELTA TOTAL	−272.2354	12.1577	1.2158

**Table 6:** Calculations of the Poisson Boltzmann equations are carried out utilizing sander’s internal PBSA solver. Each value is given in kcal/mole.

Poisson Boltzmann			
Complex:			
Energy Component	Average	Std. Dev.	Err. of Mean
VDWAALS	−13,331.9017	51.5473	5.1547
EEL	−113,422.7858	113.5974	11.3597
EPB	−18,084.3392	74.7642	7.4764
ENPOLAR	324.2708	0.9841	0.0984
G gas	−126,754.6874	115.0917	11.5092
G solv	−17,760.0684	74.5556	7.4556
TOTAL	−144,514.7558	91.3773	9.1377
Receptor:			
Energy Component	Average	Std. Dev.	Err. of Mean
VDWAALS	−10,091.7946	45.7379	4.5738
EEL	−81,782.776	118.6831	11.8683
EPB	−17,279.2394	91.9939	9.1994
ENPOLAR	256.9962	0.7679	0.0768
G gas	−91,874.5705	119.4938	11.9494
G solv	−17,022.2432	91.7489	9.1749
TOTAL	−108,896.8137	82.893	8.2893
Ligand:			
Energy Component	Average	Std. Dev.	Err. of Mean
VDWAALS	−2773.0544	20.5908	2.0591
EEL	−27,196.8614	85.3765	8.5376
EPB	−5357.5329	61.3195	6.132
ENPOLAR	120.0538	0.6479	0.0648
G gas	−29,969.9158	83.196	8.3196
G solv	−5237.4791	61.554	6.1554
TOTAL	−35,207.3949	46.5701	4.657
Differences (Complex-Receptor—Ligand)			
Energy Component	Average	Std. Dev.	Err. of Mean
VDWAALS	−467.0527	10.8948	1.0895
EEL	−4443.1483	64.8796	6.488
EPB	4552.4332	57.0836	5.7084
ENPOLAR	−52.7792	0.5371	0.0537
EDISPER	0	0	0
DELTA G gas	−4910.2011	62.5925	6.2592
DELTA G solv	4499.654	56.8086	5.6809
DELTA TOTAL	−410.5471	14.1814	1.4181

**Table 7:** Hotspot residues from TLR4 and Vaccine ensemble highly contributes to complex stabilization.

GB						PB					
Total		Sidechain		Backbone		Total		Sidechain		Backbone	
MET15	−2.08462	MET15	−2.40965	PHE237	−1.06161	MET15	−1.44671	MET15	−1.60689	SER60	−0.77127
GLU16	−1.17813	GLU16	−1.05225	LEU782	−1.66875	GLU16	−2.13704	GLU16	−1.92609	THR84	−0.8626
ASP34	−5.04397	ASP34	−5.29744	LEU783	−1.58457	ASP34	−5.13311	ASP34	−4.97831	GLY85	−0.74245
PHE37	−5.19558	SER36	−1.51823	PHE842	−1.14768	PHE37	−3.32421	PHE37	−3.20194	PHE237	−1.31058
ASP58	−1.43186	PHE37	−4.89467	LYS981	−1.69243	ARG61	−4.5124	ARG61	−4.35588	ARG238	−1.8331
ARG61	−3.40484	ASP58	−1.70757	TYR982	−0.15898	VAL108	−1.37047	VAL108	−1.32842	LEU782	−2.03181
THR84	−2.70391	ARG61	−4.11754	ASP984	−0.10635	HIE133	−3.00269	HIE133	−2.95278	LEU783	−1.1302
VAL108	−1.40956	THR84	−2.12421	SER995	−0.10725	ASP155	−5.04159	ASP155	−4.86819	PHE842	−1.28279
HIE133	−2.04865	VAL108	−1.38103	ASN996	−0.05226	LYS204	−2.44477	LYS204	−2.36699	ARG843	−1.5378
ASP155	−6.43927	HIE133	−2.03865	Residue		ARG208	−2.46066	ARG208	−2.46258	LYS981	−1.28934
LYS204	−1.98953	ASP155	−6.65367			ARG231	−5.28115	ARG231	−5.18781		
ARG208	−1.22748	LYS204	−2.1196			PHE237	−4.21487	PHE237	−2.90432		
ARG231	−4.5883	ARG208	−1.62838			ARG238	−8.6338	ARG238	−6.80086		
VAL233	−0.92789	ARG231	−4.66365			ASN239	−3.62178	ASN239	−3.18728		
PHE237	−5.58086	VAL233	−1.0402			ARG263	−2.76684	ARG263	−2.66258		
ARG238	−7.69397	PHE237	−4.51921			TYR266	−1.41979	TYR266	−1.18676		
ASN239	−3.91616	ARG238	−6.94139			VAL290	−2.20019	VAL290	−1.80646		
ARG263	−1.27341	ASN239	−3.97193			LEU393	−1.78254	LEU393	−1.92587		
TYR266	−1.13488	ARG263	−1.57907			LEU418	−2.08328	LEU418	−1.89394		
VAL290	−2.1697	TYR266	−1.14303			PHE437	−3.73036	PHE437	−3.53362		
LEU393	−2.0549	VAL290	−2.03742			MET620	−2.2787	MET620	−2.80336		
LEU418	−2.06666	LEU393	−2.26239			GLU621	−3.29011	GLU621	−3.11259		
PHE437	−4.37701	PHE414	−1.07004			ASN637	−3.3685	ASN637	−3.3096		
MET620	−3.03892	LEU418	−2.17538			ASP639	−5.13609	ASP639	−5.00157		
GLU621	−1.5327	PHE437	−4.36564			PHE642	−3.84124	PHE642	−3.71211		
ASN637	−3.12	MET620	−3.41881			ASP663	−1.4491	ASP663	−0.92414		
ASP639	−4.40471	GLU621	−1.50262			ARG666	−3.2697	ARG666	−3.41851		
PHE642	−5.54916	ASN637	−3.09983			VAL713	−1.93046	VAL713	−1.73128		
ASP663	−2.19012	ASP639	−4.64608			GLU714	−1.81689	GLU714	−1.58249		
SER665	−1.68462	SER641	−1.18362			LYS732	−5.19682	LYS732	−5.02788		
ARG666	−2.60525	PHE642	−5.27163			HIE738	−2.23817	HIE738	−1.90826		
THR689	−2.49328	ASP663	−2.32401			LEU782	−3.40719	LEU782	−1.37525		
VAL713	−1.62158	SER665	−2.22498			ARG806	−10.0732	ARG806	−9.55822		
GLU714	−3.33969	ARG666	−3.3758			HIE808	−1.15354	HIE808	−0.93046		
LYS732	−3.27881	THR689	−2.1897			HIE835	−1.17941	HIE835	−1.06968		
HIE738	−1.19432	VAL713	−1.71425			PHE842	−4.72342	PHE842	−3.44065		
HIE758	−1.06866	GLU714	−2.94628			ARG843	−6.34089	ARG843	−4.80317		
LEU782	−3.33685	LYS732	−3.87176			ASN844	−4.46346	ASN844	−4.10789		
LEU783	−2.21718	HIE738	−1.20185			ARG868	−1.40767	ARG868	−1.24233		
ARG806	−7.8341	HIE758	−1.10525			TYR871	−1.50897	TYR871	−1.29594		
HIE808	−2.951	LEU782	−1.66809			VAL895	−1.67395	VAL895	−1.68745		
HIE835	−1.78933	ASN784	−1.06739			PHE956	−2.18221	PHE956	−2.07051		
PHE842	−6.00546	ARG806	−7.91147			LYS981	−1.76858	LYS981	−2.28329		
ARG843	−6.33914	HIE808	−3.27018			TYR982	−1.39534	TYR982	−1.03322		
ASN844	−5.59359	ARG813	−1.28889			ASN996	−2.69019	ASN996	−2.59746		
ARG868	−1.71646	HIE835	−1.95128			LEU998	−1.85412	LEU998	−1.80163		
ALA870	−1.02429	PHE842	−4.85791								
TYR871	−1.17867	ARG843	−5.3531								
VAL895	−2.11224	ASN844	−5.27324								
HIE913	−1.32297	ARG868	−2.16871								
THR936	−1.09677	TYR871	−1.13235								
PHE956	−2.67546	VAL895	−1.88138								
LYS981	−2.23869	HIE913	−1.44291								
TYR982	−2.93645	ARG934	−1.00448								
ASN996	−3.03567	PHE956	−2.74494								
LEU998	−2.06441	TYR982	−2.7774								
		ASN996	−2.98316								
		LEU998	−2.14224								

## Data Availability

All data for this study are contained within the manuscript.

## Abbreviations

CL (cutaneous leishmaniasis), TLR-4 (toll-like receptor-4), VL (visceral leishmaniasis), MCL (muco-cutaneous leishmaniasis), AmB (amphotericin B), LPG (lipophosphoglycan), GP (glycoproteins), ACC (auto cross-covariance), CTL (cytotoxic T-cells), JCat (Java Codon Adaptation), RMSD (root mean square deviation), RMSF (root mean square fluctuations), LBL (linear B-lymphocytes), CAI (codon adaptation index), HSPs (Heat shock proteins).
